# Supplementary material for: Postprandial response of leptin and adiponectin to standardized high-carbohydrate and high-fat meals in adults: A cross-sectional study
Source: PLoS One. 2026 May 18;21(5):e0349380. doi: 10.1371/journal.pone.0349380 (PMC13183211; doi:10.1371/journal.pone.0349380)
Supplement: S1 Table — (DOCX) [file pone.0349380.s001.docx]

**SUPPORTING INFORMATION**

| Meal type | Macronutrient distribution  (% energy) | Food items and quantities |
| --- | --- | --- |
| High-carbohydrate, low-fat | 60% carbohydrate, 20% fat, 20% protein | Ham sandwich: white bread (2 slices), mayonnaise (7 g), turkey ham (53 g); corn cereal (30 g) with skim milk (240 mL); packaged orange juice (240 mL). calories: 570.2 |
| High-fat, low-carbohydrate | 50% fat, 30% carbohydrate, 20% protein | Ground beef (75 g) cooked with canola oil (10 mL); packaged corn tostadas (Salmas, 18 g); whole milk (240 mL); packaged orange juice (120 mL). calories: 570.6 |
| Standardized beverage (237 ml) | 63.4% carbohydrate, 22% fat, 14.6% protein | Carbohydrates (39 g), protein (9g), fat (6g). calories:246 |

**S1 Table: Standardized meals composition.**
