# Supplementary material for: Postprandial response of leptin and adiponectin to standardized high-carbohydrate and high-fat meals in adults: A cross-sectional study
Source: PLoS One. 2026 May 18;21(5):e0349380. doi: 10.1371/journal.pone.0349380 (PMC13183211; doi:10.1371/journal.pone.0349380)
Supplement: S2 Table — (DOCX) [file pone.0349380.s002.docx]

| Time (min) | Carbohydrate-rich meal  (mean ± SD, ng/mL) | Fat-rich meal  (mean ± SD, ng/mL) | p-value |
| --- | --- | --- | --- |
| 0 | 30.75 ± 23.27 | 32.03 ± 23.33 | 0.731 |
| 60 | 28.70 ± 23.47 | 28.83 ± 22.21 | 0.972 |
| 120 | 29.03 ± 23.08 | 26.73 ± 19.96 | 0.504 |
| 360 | 30.56 ± 23.36 | 27.25 ± 20.14 | 0.341 |

**S2 Table. Postprandial leptin cooncentrations by meal type.**

All values are expressed as (mean ± SD, ng/mL), and all comparisons were performed using the Wilcoxon rank-sum test.
