# Supplementary material for: Postprandial response of leptin and adiponectin to standardized high-carbohydrate and high-fat meals in adults: A cross-sectional study
Source: PLoS One. 2026 May 18;21(5):e0349380. doi: 10.1371/journal.pone.0349380 (PMC13183211; doi:10.1371/journal.pone.0349380)
Supplement: S3 Table — (DOCX) [file pone.0349380.s003.docx]

|  | Carbohydrate-rich meal | | | Fat-rich meal | | |
| --- | --- | --- | --- | --- | --- | --- |
| Time (min) | **BMI < 30**  **(n= 42)** | **BMI > 30**  **(n= 37)** | ***p*-value** | **BMI < 30**  **(n= 42)** | **BMI > 30**  **(n= 37)** | ***p*-value** |
| 0 | 20.02 ± 12.88 | 43.04 ± 26.06 | <0.001 | 21.16 ± 14.88 | 44.36 ± 25.16 | <0.001 |
| 60 | 17.32 ± 12.61 | 41.59 ± 26.09 | <0.001 | 18.39 ± 13.36 | 40.69 ± 24.36 | <0.001 |
| 120 | 18.25 ± 12.29 | 41.15 ± 25.77 | <0.001 | 16.95 ± 11.43 | 37.83 ± 21.82 | <0.001 |
| 360 | 20.04 ± 13.71 | 42.22 ± 25.48 | <0.001 | 18.24 ± 13.86 | 37.48 ± 21.40 | <0.001 |

**S3 Table. Postprandial leptin concentrations according to obesity status (n=79).**

All values are expressed as (mean ± SD, ng/mL), and all comparisons were performed using the Wilcoxon rank-sum test.
