# Supplementary material for: Postprandial response of leptin and adiponectin to standardized high-carbohydrate and high-fat meals in adults: A cross-sectional study
Source: PLoS One. 2026 May 18;21(5):e0349380. doi: 10.1371/journal.pone.0349380 (PMC13183211; doi:10.1371/journal.pone.0349380)
Supplement: S4 Table — (DOCX) [file pone.0349380.s004.docx]

| \| Time (min) \| Carbohydrate-rich meal  (mean ± SD, µg/mL) \| Fat-rich meal  (mean ± SD, µg/mL) \| *p*-value \| \| --- \| --- \| --- \| --- \| \| 0 \| 10.38 ± 4.92 \| 10.01 ± 4.66 \| 0.634 \| \| 60 \| 9.48 ± 4.50 \| 10.12 ± 4.72 \| 0.382 \| \| 120 \| 10.02 ± 4.75 \| 9.93 ± 4.51 \| 0.903 \| \| 360 \| 9.43 ± 4.64 \| 9.46 ± 4.86 \| 0.964 \| |
| --- | --- | --- | --- | --- | --- | --- | --- | --- | --- | --- | --- | --- | --- | --- | --- | --- | --- | --- | --- | --- |

**Supplementary. Table 4. Postprandial adiponectin concentrations by meal type (n=79).**

All values are expressed as (mean ± SD, µg/mL), and all comparisons were performed using the Wilcoxon rank-sum test.
