# Supplementary material for: Postprandial response of leptin and adiponectin to standardized high-carbohydrate and high-fat meals in adults: A cross-sectional study
Source: PLoS One. 2026 May 18;21(5):e0349380. doi: 10.1371/journal.pone.0349380 (PMC13183211; doi:10.1371/journal.pone.0349380)
Supplement: S5 Table — (DOCX) [file pone.0349380.s005.docx]

|  | Carbohydrate-rich meal | | | Fat-rich meal | | |
| --- | --- | --- | --- | --- | --- | --- |
| Time (min) | **BMI < 30**  **(n= 42)** | **BMI > 30**  **(n= 37)** | ***p*-value** | **BMI < 30**  **(n= 42)** | **BMI > 30**  **(n= 37)** | ***p*-value** |
| 0 | 11.57 ± 5.29 | 9.02 ± 4.13 | 0.021 | 11.23 ± 5.06 | 8.63 ± 3.78 | 0.012 |
| 60 | 10.50 ± 4.97 | 8.32 ± 3.63 | 0.031 | 11.45 ± 5.13 | 8.61 ± 3.73 | 0.007 |
| 120 | 11.37 ± 5.16 | 8.49 ± 3.76 | 0.007 | 11.20 ± 4.89 | 8.49 ± 3.57 | 0.007 |
| 360 | 10.88 ± 4.92 | 7.78 ± 3.71 | 0.002 | 10.36 ± 4.92 | 8.45 ± 4.63 | 0.081 |

**S5 Table.** **Postprandial adiponectin by obesity status and meal type.**

All values are expressed as (mean ± SD, µg/mL), and all comparisons were performed using the Wilcoxon rank-sum test.
