# Supplementary figures and images for: Postprandial response of leptin and adiponectin to standardized high-carbohydrate and high-fat meals in adults: A cross-sectional study
Source: PLoS One. 2026 May 18;21(5):e0349380. doi: 10.1371/journal.pone.0349380 (PMC13183211; doi:10.1371/journal.pone.0349380)

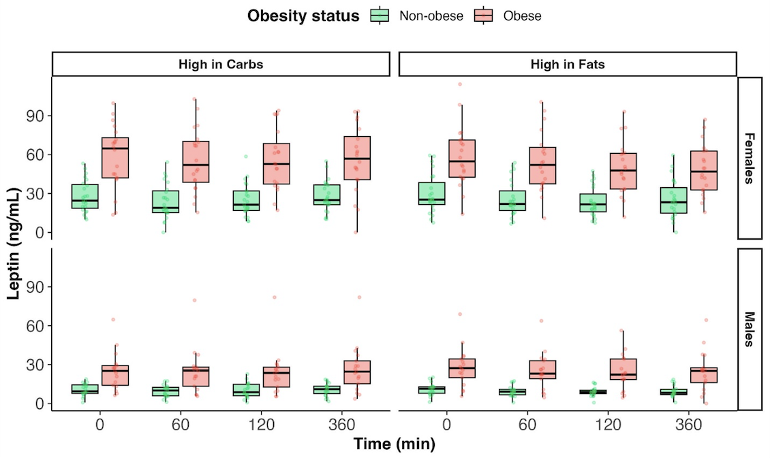

Supplement: S1 Fig — (PNG) [file pone.0349380.s006.png]

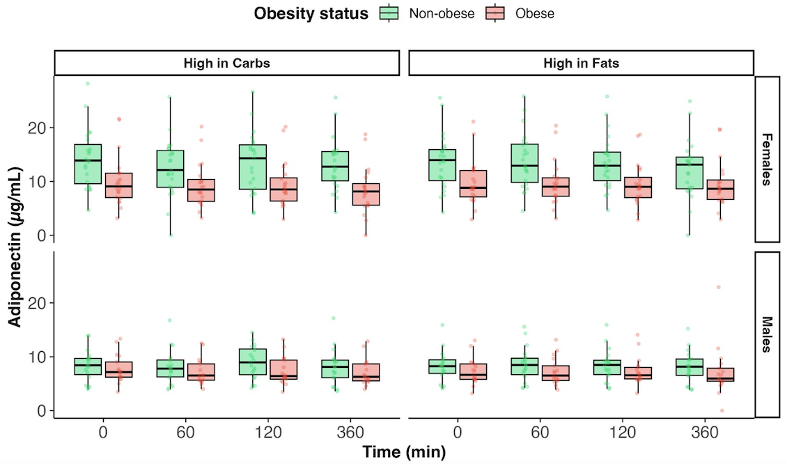

Supplement: S2 Fig — (PNG) [file pone.0349380.s007.png]
