# Supplementary material for: Postprandial response of leptin and adiponectin to standardized high-carbohydrate and high-fat meals in adults: A cross-sectional study
Source: PLoS One. 2026 May 18;21(5):e0349380. doi: 10.1371/journal.pone.0349380 (PMC13183211; doi:10.1371/journal.pone.0349380)
Supplement: Attachment — Submitted filename: Response to Reviewers.pdf [file pone.0349380.s010.pdf]

Cover Letter

Manuscript ID: PONE-D-25-57507

Title: Postprandial Response of Leptin and Adiponectin to Standardized High Carbohydrate and High-Fat Meals in Adults.

Journal: PLOS ONE

Dear Dr. Antonio-Villa

Thank you for the opportunity to revise our manuscript and for the reviewers' careful evaluation. We appreciate the constructive feedback and are pleased that the reviewers indicated that their previous comments have been adequately addressed.We carefully reviewed the manuscript once more to ensure clarity and consistency throughout the text.

We provide a response to each comment below. All changes made in the manuscript are indicated in highlighted text in the revised version, and the corresponding page numbers are provided in our responses.

We hope that the revised manuscript is now suitable for publication in *PLOS ONE*. Please let us know if any additional information is required.

Thank you again for your consideration.

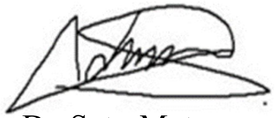

Dr. Soto-Mota

Major comments

1. Study design and crossover clarification

The manuscript states that participants completed two standardized mixed-meal tolerance tests with different macronutrient compositions. However, it is not fully clear whether the study followed a randomized crossover design or whether the order of meal administration was fixed.

Please clarify:

a：Whether meal order was randomized or counterbalanced.

Response: We thank the reviewer for this comment. All participants completed two standardized mixed-meal tolerance tests with different macronutrient compositions (high-carbohydrate and high-fat). The order of meal administration was randomly assigned for each participant to reduce potential order effects. This information has now been clarified in the Methods section. *Page 7*

b：The washout period between test days.

Response: Participants completed the two mixed-meal tolerance tests on separate visits within the same week, with an interval of 1 to 7 days between visits. Since the outcomes assessed were acute postprandial responses, a formal washout period was not implemented. *Page 7*

c: Whether potential order or carryover effects were assessed or controlled for.

Clarification is important because postprandial hormonal responses may be influenced by prior dietary exposure.

Response: Because of the short-term nature of the outcomes, the risk of carryover effects from the previous meal challenge was considered minimal. *Page 7*

2. Interpretation of meal composition effects

While the mixed-effects models identify meal composition as a significant predictor of leptin trajectories, pointwise comparisons between meals at individual time points were largely non-significant.

The Discussion should more clearly distinguish between:

a:Statistical significance in trajectory modeling versus

b:Lack of differences at discrete postprandial time points.

At present, the manuscript occasionally implies stronger meal effects than are directly supported by the pairwise comparisons. A more nuanced interpretation would improve scientific rigor and transparency.

Response: The mixed-effects models evaluate the overall postprandial trajectory across all time points simultaneously, offering greater statistical power to detect gradual, sustained effects. In contrast, the pointwise Wilcoxon comparisons test for differences at each individual time point in isolation, without borrowing information across the entire time series. The lack of significant pairwise differences at discrete time points therefore does not contradict the model-based results; it reflects the inherently lower power of single-timepoint comparisons to detect gradual trends. We have revised the Discussion to make this distinction explicit and to ensure that language throughout is consistent with the level of evidence provided by each analytical approach. Specifically, we have softened statements that implied stronger meal effects than were directly supported by the pairwise comparisons.

Change made: Added a paragraph in the Discussion: "It is important to distinguish between these two levels of inference: the mixed-effects models capture overall trajectories across all time points simultaneously, whereas pointwise Wilcoxon comparisons assess differences at each individual time point in isolation. The absence of significant pairwise differences at discrete time points does not contradict the model-based findings; rather, it reflects the greater statistical power of trajectory modeling to detect gradual, sustained effects that may not reach significance at any single time point." Additionally, the phrase 'shaped the overall hormonal response' was revised to 'shaped the overall leptin response' to avoid implying a global effect on adiponectin that was not observed. *Page 15.*

3. Adiponectin findings and model interpretation

For adiponectin, the mixed-effects model indicates that meal composition and BMI-by-meal interactions were not significant predictors, yet the Discussion suggests a modest decline after the high-fat meal.

Please clarify whether this decline reflects:

a :A statistically supported trajectory effect, or

b:A descriptive trend without formal statistical support.

If the latter, the language should be softened to avoid overinterpretation.

Response: Thank you, the comment is correct. The mixed-effects model did not identify meal composition or the BMI × meal interaction as significant predictors of adiponectin trajectories. The observed decline after the high-fat meal is a descriptive trend visible in the figures and consistent with prior literature, but it is not formally supported by the statistical model. We have revised the relevant sentences in the Discussion to make this explicit, with phrasing that clearly frames it as a descriptive, non-significant observation.

Change made: The phrase "The modest decline in adiponectin observed after the high-fat meal" was revised to "The descriptive trend toward lower adiponectin concentrations after the high-fat meal (which did not reach statistical significance in the mixed-effects model)." Similarly, "adiponectin concentrations exhibited only modest time-related changes, consistent with the mixed-model results" was revised to "a modest, non-significant time-related trend, consistent with the absence of a significant meal or BMI×meal effect in the adiponectin mixed-effects model." *Page 15.*

4. Data availability statement

The Data Availability section indicates that data are part of Mexico’s BIOMEM biobank and are available upon meeting criteria for access.

Please specify:

a:Whether aggregated or de-identified data underlying the figures and tables (e.g., summary statistics) can be made publicly available.

b:The process and contact information for requesting access.

This clarification is necessary to ensure compliance with PLOS ONE’s data availability requirements.

Response:

We appreciate the reviewer attention to the PLOS Data Availability Policy. To clarify data access procedures, we have revised the Data Availability Statement to specify that the dataset belongs to Mexico's Biobank for Medical Diseases (BIOMEM) and that access is subject to institutional ethical and legal restrictions regarding participant privacy. For this reason, neither individual-level nor aggregated datasets can be publicly available. However, de-identified data supporting the figures and tables can be provided to qualified researchers upon reasonable request and approval by the institutional Ethics Committee. Researchers who

meet the criteria for access to confidential data can request it through the Ethics Committee of the Instituto Nacional de Ciencias Médicas y Nutrición Salvador Zubirán. Data requests can be sent to the corresponding author at adrian.sotom@incmnsz.mx or to the Biobanco Mexicano de Enfermedades Metabólicas at biomem@incmnsz.mx, who will coordinate review with the institutional review board. The institutional research ethics committee can be contacted at comite.etica.investigacion@incmnsz.mx. *Page* 1 and 2.

Minor comments

Statistical reporting

Please specify whether residual diagnostics were examined for the linear mixed-effects models.

Clarify whether time was modeled as a continuous or categorical variable.

Response: We confirm that residual diagnostics were conducted for both models. Visual inspection of histograms and standard Q-Q plots of the residuals supported the assumption of approximate normality in both the leptin and adiponectin models. Time was modeled as a continuous variable (in minutes), allowing the model to estimate a single average rate of change in adipokine concentration per minute across the postprandial period. Both of these details were already present in the Statistical Analysis section; however, we have reorganized and expanded the relevant sentences to make them more explicit and easier to locate.

Change made: Added to the Statistical Analysis section: "In the linear mixed-effects models, time was modeled as a continuous variable (in minutes), allowing the model to estimate a single average rate of change in adipokine concentration per minute across the postprandial period. Model assumptions were evaluated through residual diagnostics, including visual inspection of histograms and standard Q-Q plots of the residuals, which supported the assumption of approximate normality." *Page 9*

Terminology

In several places, “stable” is used without an explicit statistical reference. Consider replacing with “did not change significantly” for precision.

Response: We have revised the manuscript to replace this term with “did not change significantly” where appropriate to improve clarity and precision.

Figures

Figures are clear and informative. However, the color gradient representing BMI may be difficult to interpret for readers with color vision deficiencies. Consider whether an alternative or simplified representation is feasible.

Response: We have replaced the original yellow-to-red gradient for BMI with the viridis color scale (option D), which is perceptually uniform and designed to remain interpretable across the most common forms of color vision deficiency. Additionally, the green/red color pair previously used to distinguish obesity groups has been replaced with the Okabe-Ito palette (blue #0072B2 for non-obese; orange/amber #E69F00 for obese), which is widely recommended for scientific figures requiring color vision accessibility (Okabe & Ito, 2002; Wong, Nature Methods, 2011). The revised figures have been re-exported as TIFF files at 300 DPI with LZW compression, meeting PLOS ONE technical requirements.

Change made: All four figures (1A–1D) were regenerated using the viridis color scale for BMI trajectories and the Okabe-Ito palette for obesity group comparisons.

Typographical and stylistic issues

Minor grammatical inconsistencies remain (e.g., spacing, punctuation around units). A final careful proofreading is recommended.

Response: The manuscript has been carefully proofread, and minor typographical and stylistic inconsistencies (including spacing and punctuation around units) have been corrected throughout the text.

7. PLOS authors have the option to publish the peer review history of their article (what does this mean?). If published, this will include your full peer review and any attached files. Yes

If you choose “no”, your identity will remain anonymous but your review may still be made public.
