# Supplementary material for: Postprandial response of leptin and adiponectin to standardized high-carbohydrate and high-fat meals in adults: A cross-sectional study
Source: PLoS One. 2026 May 18;21(5):e0349380. doi: 10.1371/journal.pone.0349380 (PMC13183211; doi:10.1371/journal.pone.0349380)
Supplement: Attachment — Submitted filename: Cover Letter, Jan-20-26- Final.docx [file pone.0349380.s009.docx]

**RESPONSE TO REVIEWERS**

**Title:** **Postprandial Response of Leptin and Adiponectin to Standardized High Carbohydrate and High-Fat Meals in Adults.**
**Manuscript ID:** PONE-D-25-57507

**Dear Dr. Antonio-Villa,**

We sincerely appreciate the opportunity to revise and improve our manuscript entitled **“**Postprandial Response of Leptin and Adiponectin to Standardized High Carbohydrate and High-Fat Meals in Adults,**”** and for the time and effort invested by you and the reviewers in evaluating our work.

We have carefully reviewed all your comments and those of the reviewers. Below, we provide a response to each comment. To facilitate their review, all editorial-team comments are displayed in red. Our manuscript has been revised accordingly, and all changes made in the enclosed manuscript are indicated in highlighted text in the revised version. To facilitate reviewing these changes, we added the corresponding page numbers in our responses, too.

We hope this current version of our manuscript now meets the standards to be eligible for publication in your journal. Nonetheless, we would be happy to provide any additional information if required. Thank you once again for the time, effort, and consideration granted to our submission.

**Dr. Adrian Soto-Mota, MD, PhD, FACP.**

*Chief of the Metabolic Diseases Research Unit*

*National Institute of Medical Science and Nutrition Salvador Zubirán*

[*adrian.sotom@incmnsz.mx*](mailto:adrian.sotom@incmnsz.mx)*,*

*phone: +5255-5487-0900, ext: 6319*

**Editorial Comment:
*If applicable, we recommend that you deposit your laboratory protocols in protocols.io to enhance the reproducibility of your results.***

**Response:** Thank you for this recommendation. At this stage, we did not deposit our laboratory protocols in protocols.io, as the procedures used in this study are fully described in the Methods section. Any additional information regarding our methods can be obtained by emailing the corresponding author. We will adopt this suggestion in future projects.

**Journal Requirements:**

***1. Please ensure that your manuscript meets PLOS ONE's style requirements, including those for file naming.***

**Response:** We confirm that the corrected manuscript has been thoroughly reviewed and revised to fully adhere to PLOS ONE’s style and formatting standards, including file naming conventions. The new version of our manuscript was formatted using the official PLOS ONE style templates for the main body, title page, authors, and affiliations.

***2. We note that the grant information you provided in the ‘Funding Information’ and ‘Financial Disclosure’ sections does not match. When you resubmit, please ensure that you provide the correct grant numbers for the awards you received for your study in the ‘Funding Information’ section.***

**Response:** Thank you for the comment.  The Funding Information and Financial Disclosure sections have been reviewed and corrected to ensure consistency.

***3. Please note that your Data Availability Statement is currently missing the DOI/accession number of each dataset OR a direct link to access each database. If your manuscript is accepted for publication, you will be asked to provide these details on a very short timeline. We therefore suggest that you provide this information now, though we will not hold up the peer review process if you are unable.***

**Response:** Thank you for pointing this out. Since this study is part of the Mexican Biobank for Metabolic Diseases (BIOMEM), we are not allowed to make the datasets immediately available. The Data Availability Statement has been revised to reflect this.

***4. Please include captions for your Supporting Information files at the end of your manuscript, and update any in-text citations to match accordingly. Please see our Supporting Information guidelines for more information:*** [**http://journals.plos.org/plosone/s/supporting-information**](http://journals.plos.org/plosone/s/supporting-information)***.***

**Response:** Captions for all Supporting Information files have now been corrected at the end of the manuscript, and all corresponding in-text citations have been revised and modified to ensure consistency with the Supporting Information files. Page 20.

***5. If the reviewer comments include a recommendation to cite specific previously published works, please review and evaluate these publications to determine whether they are relevant and should be cited. There is no requirement to cite these works unless the editor has indicated otherwise.***

**Response:** Thank you for this clarification. All publications suggested by the reviewers have been carefully evaluated and, since they are relevant indeed, added to the manuscript where appropriate, in accordance with the reviewers’ guidance. Page 21.

**Additional Editor Comments:**

***Please include a STROBE checklist that best adheres to the study design the authors used.***

**Response:** A revised version of the manuscript addressing all points raised during the review process has been submitted. In addition, an appropriate STROBE checklist for the study design has been included as Supporting Information.

***1. The phrase “The institutional ethics committee approved the study.” is redundant, considering that the authors previously stated: “The institutional Ethics in Human Research Committee assessed and approved the protocol (reference number 3722).”***

**Response:** We amended our text accordingly. Page 6.

***2. A potential limitation of the study would be the exclusion of individuals living with diabetes, as this population also tends to have differential secretion of leptin and adiponectin, particularly in the elderly population (PMID: 33411633). This could prompt collider bias given the combined prevalence of diabetes and obesity in the Mexican population.***

**Response:** Thank you for this insightful comment. The exclusion of subjects with previously diagnosed diabetes was a prior design decision to characterize postprandial leptin and adiponectin responses in adults without chronic metabolic disease beyond overweight or obesity. This approach allowed us to reduce metabolic heterogeneity and potential confounding related to glucose-lowering treatments and diabetes duration. Nonetheless, we agree that this exclusion criterion may limit the generalizability of our findings and could introduce collider bias, and we included these points in our limitations section. Page 19.

***3. Leptin seems to have a skewed distribution (given the high SD presented in Table 1). In this regard, did the residuals of the mixed-effects linear regression model fulfill a normal distribution?***

**Response:** Thanks for this important statistical point. Although leptin concentration showed a right-skewed distribution, model assumptions were evaluated based on the mixed-effects model residuals. Residual diagnostics, including histograms and Q-Q plots, indicated approximate normality, with only minor deviations at the extremes. Therefore, the use of mixed-effects linear regression models was considered appropriate.

**4. *Consider including the 95% confidence intervals of the coefficients reported in Table 2 and Table 3. These could be obtained using the Wald approximation or a semiparametric bootstrap. The lme4 R package further expands these approximations*:** <https://cran.r-project.org/web/packages/lme4/vignettes/lmer.pdf> -

**Response:**

Thank you for the suggestion. We have added 95% confidence intervals for all fixed-effect coefficients to Tables 2 and 3, using the Wald approximation implemented in the lme4 package. The tables had been updated accordingly.

***5. Additionally, please specify the fixed effects (time, sex, BMI, meal type) and random effects (individual) used in the models.***

**Response:** Thank you for the suggestion. We have now explicitly specified the fixed effects (time, sex, BMI, and meal type) and the random effect (individual participant), and we have included mixed-effects models in the Statistical Analysis section. Page 8.

**Minor comments**

***In Table 2, please consider changing the labels to “Men” instead of only “Sex” and “Low-carbohydrate meal” instead of “Meal type.”***

**Response:** Thank you. We have replaced “Sex” with “Men” and “Meal type” with “Low-carbohydrate meal” in tables 2 and 3.

***In figures, please be consistent with the labels “Men” and “Women.”***

**Response:** Thank you. All figures have been reviewed and updated with consistent labels “Men” and “Women.”

***Table 4 was not appended in the document.***

**Response:** Thank you. This was a numbering error, which has now been corrected, and all table numbering has been updated accordingly.

***I believe there is a typo in lines 200 and 240.***

**Response:** Thank you. Lines 200 and 240 were carefully reviewed, and the likely typographical issues were corrected in the revised manuscript.

**Major Comments:**

***Data Presentation and Interpretation Issues:***

***Confusing Citations of Main and Supplementary Tables: The text repeatedly misquotes Main Tables 2 and 4 (which are the primary tables presenting mixed-effects model results) when discussing pointwise comparisons of postprandial leptin and adiponectin. The detailed data for these pointwise comparisons should be presented in Supplementary Tables 2, 3, 4, and 5. For example, lines 174-175: "When comparing meal types, no significant differences in leptin concentrations were observed at any postprandial time point between the high-carbohydrate and high-fat meals (Table 2)." should be corrected to cite Supplementary Table 2. Please carefully check and correct all table citations in the text to ensure the purpose and citation logic of main and supplementary tables are clear.***

**Response:** Thank you for this observation. We have revised all table citations throughout the manuscript and corrected them.

***Detailed Explanation of Mixed-Effects Models (Tables 2 and 3): This remains a core issue, especially given that mixed models are a primary strength of this study. The meaning of their coefficients must be precisely elucidated in the main text:***

**Response:** We agree that a clear interpretation of mixed-effects model coefficients is essential. We have revised this section and explained the meaning of each β coefficient in Tables 2 and 3. We have carefully reviewed all results to ensure full consistency between the main text and the main and supplementary tables. In addition, errors related to the specification of the meal reference category in the mixed-effects models were identified and corrected in Tables 2 and 3. Pages 10-11.

***β-value for "Sex": Please clarify whether it represents an average concentration difference (with females as reference) or a difference in time trends. Based on Table 1 and the negative β-value, this appears to represent an average level difference for males compared to females, which should be explicitly stated in the text.***

**Response:** The β coefficient for sex represents an average difference in adipokine concentrations between men and women, with women specified as the reference category, and does not reflect a difference in time trends. The negative β value, therefore, indicates lower overall postprandial leptin and adiponectin concentrations in men compared with women, independent of time and meal type. We have revised the Results section accordingly.

***β-value for "Meal Type" (Leptin: 5.93, Adiponectin: -5.0): Please clearly explain its meaning. Does it represent the average concentration difference for the high-carbohydrate meal relative to the high-fat meal (reference level), or a difference in their overall postprandial trajectories? Given that supplementary tables show no significant pointwise differences, but the mixed model indicates meal type as a significant predictor, this distinction is crucial and requires clear clarification.***

**Response:** The β coefficient for *meal type* in the mixed-effects models represents an average difference in adipokine concentrations across the entire postprandial period, adjusted for time, sex, BMI, and their interaction, with the high-carbohydrate meal specified as the reference category.

For leptin, the β-value indicates that, on average, postprandial leptin concentrations were higher following the high-fat meal compared with the high-carbohydrate meal, independent of time trends. This coefficient does not reflect differences in time-specific trajectories. The absence of significant pointwise differences between meals in the supplementary tables reflects the use of nonparametric comparisons at individual time points, whereas the mixed-effects model estimates an overall adjusted mean difference across measurements.

***Interpretation of "BMI : Meal Type" Interaction Term: For leptin, this interaction term is significant (β = -0.23, p = 0.005), which supports the conclusion that "obese participants exhibited a significantly greater postprandial decline after the high-fat meal." Please elaborate on the biological significance of this interaction effect in the results and discussion, specifically, how the difference in leptin response between the two meal types changes for every one-unit increase in BMI. For adiponectin, this interaction term is not significant, indicating no differential effect of BMI on different meal types, which should also be stated in the text.***

**Response:** We have revised both the results and discussion sections to explicitly interpret the BMI x meal type interaction. For leptin, the significant negative interaction term (*β = -0.23, p = 0.005)* indicates that for each 1 unit increase in BMI, the difference in postprandial leptin concentrations between the high-fat and high-carbohydrate meals decreases by 0.23 ng/mL, reflecting a greater postprandial decline in leptin after the high-fat meal as adiposity increases. For adiponectin, we explained that the BMI x meal interaction was not significant, indicating no differential effect of BMI on adiponectin responses across meal types.

***Detailed Interpretation of Figure 1: Figure 1 clearly presents the results, but the description and interpretation of Figure 1 in the main text still need strengthening. Please elaborate on the key trends and differences presented in each panel of Figure 1 in the results section and connect them with the findings from the mixed-effects models (especially interactions). For example, how can the trend that "obese participants exhibited a significantly greater postprandial decline after the high-fat meal" be observed from Figure 1?***

**Response:** The results section was revised to explicitly describe the key trends observed in each panel and to connect them with the mixed-effects model results, particularly the significant interaction between BMI and type of meal for leptin. In addition, Figure 1 was carefully reviewed, and the panel order was slightly modified to improve clarity, with men consistently shown on the left and women on the right across all panels. Pages 12-13

**Methodological Details:**

***Inconsistent Description of Study Design: Line 101 of the manuscript still describes it as "This cross-sectional study…", which contradicts the actual "crossover design with repeated measures." Please ensure the study design type is corrected to an accurate description and maintained consistently throughout the manuscript.***

**Response:** Thank you. We have revised the manuscript to ensure the cross-sectional design is consistently and clearly described throughout the text.

***Washout Period and Meal Order: This information is still missing. A crossover study design requires an adequate washout period to prevent carryover effects between interventions. Please clearly state the interval between the two MMTTs (washout period) in the methods and justify its adequacy. Additionally, please state whether the order of meal administration was randomized and whether any sequence or period effects were assessed.***

**Response:**  Thank you for this comment. Participants completed two test visits within the same week because both interventions were designed as acute postprandial challenges, and to minimize changes in habitual diet and lifestyle that could confound results. We included these details in the methods section. Page 7.

***Detailed Information on Standardized Dinner: The methods section mentions that "all participants consumed a standardized dinner the evening before each test day." While Supplementary Table 1 provides details of the test meals, the composition of the standardized dinner (macronutrients, calories, specific foods) is equally important for controlling the metabolic state before testing. Please provide these details in the methods or supplementary materials.***

**Response:** We have now provided detailed information on the standardized dinner, including total energy content and macronutrient composition, in the Methods section (page 8) and Supplementary Table 1.

***Further Clarification of Statistical Analysis:***

***Clear Distinction Between Wilcoxon and Mixed Models: The manuscript needs to clarify the respective roles of the Wilcoxon pointwise comparisons (Supplementary Tables 2-5) and the linear mixed-effects models (Main Tables 2-3). Wilcoxon tests show differences at specific time points, while mixed models capture overall longitudinal trajectories and interactions. The discussion must clearly reconcile the results of these two analytical approaches, explaining why single time points might not show significant differences, while the overall trajectory is significantly influenced by predictors.***

**Response:** We have revised the Statistical Analysis and Discussion sections to explicitly distinguish the roles of the Wilcoxon pointwise comparisons and the linear mixed-effects models. We now clarify that Wilcoxon tests assess between-group differences at individual time points, whereas mixed-effects models capture overall longitudinal trajectories and interactions across the postprandial period. We further explain that the absence of significant differences at single time points does not contradict significant effects on overall trajectories identified by the mixed-effects models.

***Random Slopes for Time: The current model only includes "random intercepts for participants." Please consider whether "random slopes for time" should be included to account for individual differences in postprandial trends. If not included, please provide a clear justification.***

**Response:** The inclusion of subject-level random intercepts captured substantial between-individual variability in baseline adipokine concentrations, as supported by the high intraclass correlation coefficients. Random slopes for time were not included, as the primary objective was to estimate population-level postprandial trends and interaction effects, and the random-intercept models provided adequate fit while maintaining model parsimony and interpretability.

***Utilization of R² Values: The marginal R² and conditional R² values of the model have been reported, indicating good explanatory power. Please utilize these R² values more deeply in the discussion to elaborate on the extent to which the selected predictors (sex, BMI, meal composition) explain the variance in leptin and adiponectin levels.***

**Response:** Thank you. We expanded the results section to elaborate on their interpretation. It now explains variability in leptin and adiponectin levels, as well as the additional contribution of between-individual variability captured by random effects. Page 11.

**Elaboration of Results and Discussion:**

***Integration and Logical Coherence of Core Findings: There is a contradiction between the conclusions in the abstract and results section regarding "postprandial leptin and adiponectin gradually decreased after a high-fat meal and remained stable after a high-carbohydrate meal" and "meal composition is a significant predictor," versus the statement in the main text (lines 174-175) that "When comparing meal types, no significant differences were observed at any postprandial time point." The authors must clearly explain this apparent contradiction in the results and discussion. For instance, emphasize that the mixed model captures trends over time or overall effects, while pointwise comparisons may not show significant instantaneous differences due to smaller effect sizes or insufficient statistical power.***

**Response:** Thank you. We now explicitly state in the Results and Discussion that while single postprandial time points did not show significant differences between meals, the mixed-effects models showed that meal composition significantly influenced leptin trajectories over time, particularly through the BMI × meal interaction.

***Deepening Mechanistic Insights and Expanding References: The discussion section lacks in-depth exploration of potential mechanisms for the observed phenomena (especially the decrease in leptin and adiponectin after a high-fat meal). Please expand this section, incorporating existing literature to discuss possible physiological mechanisms, such as the effects of different macronutrients on insulin, glucose, gut hormones (e.g., CCK, GLP-1), as well as fatty acid types, inflammatory responses, etc. The current number of 13 references is relatively small; it is recommended to supplement with more comprehensive and relevant literature to support the depth of the background and discussion.***

**Response:** We amended the relevant section accordingly. Page 16.

***Clinical Significance and "Leptin Resistance": The discussion states that "a reduction in leptin, particularly in obese individuals, may impair satiety signals and contribute to excessive energy intake." Please further link this finding to the concept of "leptin resistance," and explain how these postprandial dynamics offer new perspectives on energy imbalance in obesity.***

**Response:** We amended the relevant section accordingly. Page 16.

**Language and Formatting:**

***Language Correction: In the abstract, "Contrastingly, stable and did not change significantly after the high-carbohydrate meal" is still missing a subject (e.g., "their concentrations remained stable"). Please carefully check the entire manuscript to ensure fluent language, accurate expression, and correct all grammatical and spelling errors (e.g., "produces" in line 342 "All plots were produces using ggplot2." should be "produced").*** ***Acronym Handling: Ensure all acronyms are defined upon their first appearance. "LCHF" in the list of acronyms is not directly relevant to the meal descriptions in this study; if not further discussed in the main text, it is suggested to remove it.***

**Response:** We thank the reviewer for the thorough and careful language review. We have revised the entire manuscript to improve clarity and fluency and corrected all identified grammatical and typographical errors throughout the text. We removed the acronym LCHF.

***Table Units and Legends: Please carefully check the units for adiponectin in Supplementary Tables 4 and 5, currently displayed as "(mean ± SD, ng/mL)," which is likely a typo and should be µg/mL. Ensure all tables and figure legends are clear, self-consistent, and contain complete information.***

**Response:** Leptin concentrations are reported consistently in ng/mL, and adiponectin concentrations in /mL throughout the manuscript and supplemental materials.

**Minor Comments:**

***Ethics Approval and Data Collection Dates: Lines 106-107 "Recruitment started on July 1st, 2022 and finished on July 31st, 2025" suggest the study is still ongoing. Please confirm whether data collection was completed at the time of manuscript submission. If so, please update the end date to reflect its completed status.***

***Reference Dates and Format: Dates such as "cited 2025 Sep 21" or "cited 2025 May 15" in the references are still future dates and should be corrected to actual citation or retrieval dates. PLOS One encourages the use of DOIs or PMCID.***

**Response:** We confirm that participant recruitment and data collection were completed in July 2025, prior to manuscript submission. This has been clarified in the Methods section.

***Please check the reference format to ensure full adherence to PLOS One's guidelines***

**Response:** All references have been carefully reviewed to adhere to the journal’s guidelines.

***Lean Mass Data in Table 1: Table 1 shows that average lean mass for females (49.02 ± 9.86 kg) is higher than for males (43.11 ± 9.83 kg, p=0.011), which contradicts general knowledge for the adult population. Please re-verify these data. If the data are accurate, please provide a reasonable explanation in the discussion (e.g., specific characteristics of the study population, age distribution, body composition measurement methods) or highlight it as a unique finding of this study.***

**Response:** Thank you for carefully reviewing Table 1. We identified a labeling error in which the lean mass values for men and women were inadvertently inverted. This has now been corrected in the revised version of the manuscript.

***Data and Code Availability: The manuscript submission system indicates data availability as "No - some restrictions will apply," with the explanation that data belong to a biobank and are only accessible to qualified researchers. This is an acceptable exception; please ensure this data availability statement is explicitly included in the methods section or supplementary information of the main manuscript, along with contact information or a process for obtaining the data.***

Thank you. We made this clear in the manuscript.

***Reporting Guidelines: It is recommended that the authors refer to relevant reporting guidelines, such as STROBE (for observational studies) or STROBE-nut (for nutritional studies), when preparing the revised manuscript to ensure comprehensive and transparent reporting of the research.***

Thank you. We referred to STROBE guidelines in this new submission.

***Conclusion:***

***Now, with the complete manuscript content (including Figure 1 and all tables), a comprehensive evaluation is possible. The topic explored in this study is interesting, and valuable findings have been obtained through the use of mixed-effects models. However, to meet PLOS One's rigorous publication standards, particularly regarding the precision of methodological descriptions, the clear integration and in-depth interpretation of different statistical analysis results, and the depth of discussion on potential mechanisms, comprehensive revisions are still required. Please address all the comments above upon resubmission. If these issues can be adequately resolved, I believe this manuscript will make a valuable contribution to the field.***

Thank you. We hope that the new version of our report meets the required standards to be eligible for publication in your journal.

***Reviewer #2: This article offers a novel perspective. Despite the relatively small sample size, it has preliminarily uncovered the phenomenon that leptin and adiponectin levels tend to decrease following a high - fat meal. Nevertheless, the study did not conduct an in - depth exploration of the underlying mechanisms. For instance, it remains unclear whether changes in key metabolic signals, such as postprandial insulin, free fatty acids, inflammatory factors, or gastrointestinal hormones, are involved in regulating this response. It is recommended that in the discussion section, possible pathways should be further hypothesized in conjunction with existing literature. Specifically, does high - fat intake result in a reduction in circulating levels by inhibiting leptin secretion or accelerating its clearance? Does postprandial lipemia impact the stability or detection accuracy of adiponectin? Supplementing the relevant experiments to further elucidate the aforementioned potential mechanisms would substantially strengthen the integrity and persuasiveness of the study. These mechanistic considerations will substantially enhance the scientific rigor and theoretical value of the article.***

**Response:** Thank you for your assessment. While elucidating the mechanistic insights behind the observed changes in adiponectin and leptin falls beyond the scope of our paper and reach of our methods, we think it is reasonable to say that these results provide nuance to the common perception of leptin and adiponectin as “steady state” signals. We amended the text accordingly in page 26.

***Reviewer #3:***

***Overall comments: The manuscript was well written and easy to follow. The introduction and discussion sections included pertinent information, and the study design was thorough. There were only a few comments to improve reader comprehension as outlined below:***

***It would be helpful for the authors to add bar graphs to demonstrate postprandial declines in both leptin and adiponectin between sexes and obese states to more clearly represent differences. These would be in relation to figure 1 and allow for more direct comparisons visually between the different groups.***

**Response:** Thank you. In order to complement the visual assessments of our results, we added new graphical representations to the main manuscript in the supplementary material. We chose boxplots, as this format more accurately reflects the distribution and variability of hormone concentrations during each time point.

***Table 4 is referenced in line 180 but not included in the data:***

***The reference to “Table 4” was a typographical error and has now been corrected to refer to the appropriate Supplementary Table. The manuscript has been carefully revised to ensure that all table references are accurate and consistently reported.***

**Response:** Thank you. The reference to “Table 4” was a typographical error and has now been corrected to refer to the appropriate Supplementary Table. The manuscript has been carefully revised to ensure that all table references are accurate and consistently reported.

***Reviewer #4: This study by Del Razo-Olvera and co-workers addresses the postprandial responses of two of the most paradigmatic hormones produced by adipose tissue (adipokines), central in the control of metabolic homeostasis (adiponectin) and our relationship with food (leptin), driving together, when defective, the development of lifestyle diseases. The question that they arise is, then, very important, and the study they developed, thus, pretty pertinent. However, it is not true that there is a lack of information regarding the postprandial fluctuations of these two well-known and extensively studied adipokines, or that their concentrations are unaffected by food intake. There is room for improvement in this regard, and quite a lot of very compelling studies dealing with the topic being neglected. The authors need to state crystal clear what’s supposed to be new and what’s confirmatory in their study, and leverage their results with the data of others researchers digging in the topic. Below, there are some other suggestions that the authors of this study can, hopefully, find useful, in order to boost their research results:***

***Additional points that might improve after revision:***

***1)  Some typos and mistakes have been identified along the manuscript. Please, be careful we word usage.***

**Response:** Thank you. We have carefully proofread the entire manuscript and corrected typographical, grammatical, and wording errors throughout the text to improve clarity and comprehensiveness.

**2) *Some references are missing/not very well placed/explained along the text. The authors neglect some key literature on the topic. Please, revise.***

**Response:** Thank you. We have carefully revised the manuscript to improve the placement, integration, and explanation of references throughout the text. In addition, we have incorporated additional literature relevant to postprandial leptin and adiponectin regulation.

***3) The plots provided (Fig.1c-d) are okay, but they do not really show the changes the authors claim in the text, apparently provided in supplementary tables. To better contextualising the information provided in text and overall picture, please include any relevant data as main article figures.***

**Response:** We have carefully re-evaluated Figure 1 in relation to the supplementary material and the text. The purpose of Figure 1 is to illustrate overall postprandial trajectories and group-level patterns captured by the mixed-effects models, whereas the supplementary tables present pointwise comparisons at specific time points. We have revised the figure description and interpretation to more explicitly reflect what the data visually support. Additionally, we have confirmed that all principal outcomes that rely on pointwise differences remain clearly referenced to the supplementary tables, while the main figures emphasize longitudinal trends and interactions. We believe this approach maintains coherence, avoids redundancy, and preserves the conceptual distinction between trajectory-based and pointwise analyses.

***4) Some additional markers following the lead of leptin and adiponectin in response to these two dietary inputs may help in boosting the relevance and clinical interest of this study. I am thinking about post-prandial measures of glucose, insulin and ghrelin during the time-course, see to what extent they level adiponectin and leptin fluctuations.***

**Response:** We agree that the inclusion of additional postprandial metabolic and hormonal markers, such as glucose, insulin, or ghrelin, would further enhance the clinical and mechanistic relevance of the study. However, these additional markers were not available for all participants. We acknowledge this limitation in the Discussion.

***5) Some authors have related the adiponectin-leptin ratio to meta-inflammation (e.g., PMID: 30813240, PMID: 34333093). Do the current results show anything in that line of though?***

**Response:** Thank you. The present study was not specifically designed to evaluate this ratio, and therefore, this metric was not calculated or analyzed. Nonetheless, we agree that these references are relevant and include them. Page 16.

***6) “These results highlight the relevance of dynamic postprandial phenotypes as early markers of metabolic dysfunction.”: Not sure what that means.***

**Response:** Thank you for the opportunity of expressing ourselves better. We rephrased our statement to improve its clarity. Page 20.
