# Supplementary material for: Postprandial response of leptin and adiponectin to standardized high-carbohydrate and high-fat meals in adults: A cross-sectional study
Source: PLoS One. 2026 May 18;21(5):e0349380. doi: 10.1371/journal.pone.0349380 (PMC13183211; doi:10.1371/journal.pone.0349380)
Supplement: Attachment — Submitted filename: PointbyPoint Reply.docx [file pone.0349380.s011.docx]

April 27^th^, 2026

Dear Dr. Antonio-Villa,

Thank you for the opportunity to revise our manuscript once more and for the constructive editorial comments. We have carefully addressed each point raised in your decision letter. All changes made in the revised manuscript are indicated with tracked changes, and the corresponding locations are referenced in our responses below. We hope that the revised manuscript is now suitable for publication in *PLOS ONE*. Please let us know if any additional information is required.

**Additional Editor Comments**

**1. Study design terminology**

***Editor comment:*** *The manuscript title describes the study as “cross-sectional,” whereas the cover letter refers to it as a “crossover study.” Please harmonize this accordingly.*

**Response:** We thank the Editor for noting this inconsistency. The study design is cross-sectional with repeated postprandial measurements under two standardized meal conditions; it is not a crossover trial. The manuscript consistently and correctly describes the design as “cross-sectional” throughout the text, Methods, and in alignment with the STROBE reporting guideline. The error was in the previous cover letter, which has now been corrected. The revised cover letter accompanying this resubmission uses “cross-sectional study” to match the manuscript.

**2. Reference level inconsistency between Results text and Tables 2 and 3**

***Editor comment:*** *Please carefully revise Tables 2 and 3 before publication. The reference level for meal type appears inconsistent between the Results text and the table footnotes. The Results state that women and the high-carbohydrate meal were used as reference categories, whereas the table footnotes state that “High in Fats” is the reference level.*

**Response:** We thank the Editor for drawing our attention to these issues. After careful review, we confirmed that the table footnotes are correct: the reference level for meal type in the mixed-effects models is the high-fat meal (i.e., “High in Fats”), and the reference for sex is Female. The error was in the Results text, which incorrectly stated that the high-carbohydrate meal was the reference category. We have corrected the Results text to read: “Women and the high-fat meal were used as reference categories for sex and meal type, respectively.” Additionally, we corrected the subsequent interpretation of the β coefficients. The coefficient for “Meal High-carbohydrate” (β = 5.93) now correctly refers to the high-carbohydrate meal (relative to the high-fat reference), and the interaction term (BMI × Meal High-carbohydrate) is described accordingly. The sentence about BMI-related increases being “weaker after the high-fat meal” was corrected to “weaker after the high-carbohydrate meal in comparison with the high-fat meal.”

**3. Typographical errors in the Results section**

***Editor comment:*** *In the Results, there appear to be some typos in the line: “relatively stablenon-significantly changing adiponectin concentrations.” This should be corrected to “non-significantly changing adiponectin concentrations” or “relatively stable adiponectin concentrations.”*

**Response:** Thank you for bringing this up. The phrase has been corrected to “relatively stable adiponectin concentrations,” which we believe is the clearest phrasing.

**4. Softening the leptin–satiety–energy intake claim**

***Editor comment:*** *The sentence stating that reductions in leptin “could attenuate satiety signaling and contribute to excessive energy intake” should be slightly softened, since satiety, appetite, and subsequent energy intake were not directly measured. I would suggest wording such as: “could potentially influence satiety-related pathways, although this hypothesis requires confirmation in studies directly assessing appetite and energy intake.”*

**Response:** Thank you for this suggestion. We have adopted it in both the Abstract (Conclusions) and the Discussion.

**5. Minor typographical issues**

***Editor comment:*** *Please revise minor typographical issues throughout the text, including “adipoectin,” spacing around references and punctuation, and inconsistent capitalization of “Sex,” “BMI,” and “Meal Type” when they are not table labels.*

**Response:** Thank you. We corrected the typo “adipoectin” → “adiponectin” in the Results section (adiponectin model description). We have also reviewed the manuscript for spacing around references, punctuation, and capitalization consistency. In the running text, “sex,” “BMI,” and “meal type” are now used in lowercase except when they appear as table row labels.

**6. Reference list review**

***Journal requirement:*** *Please review your reference list to ensure that it is complete and correct. If you have cited papers that have been retracted, please include the rationale for doing so in the manuscript text, or remove these references and replace them with relevant current references.*

**Response:** We have reviewed the reference list and confirmed that all 22 cited works are current, accessible, and none have been retracted. No changes to the reference list were necessary.

Thank you again for your consideration.

Adrian Soto-Mota, MD, PhD, FACP

Chief of the Metabolic Diseases Research Unit

National Institute of Medical Sciences and Nutrition Salvador Zubirán
